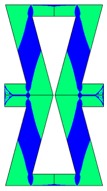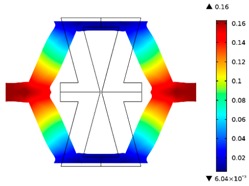# Computational Modelling of Structures with Non-Intuitive Behaviour

**DOI:** 10.3390/ma10121386

**Published:** 2017-12-04

**Authors:** Tomasz Strek, Hubert Jopek, Eligiusz Idczak, Krzysztof W. Wojciechowski

**Affiliations:** 1 Institute of Applied Mechanics, Poznan University of Technology ul. Jana Pawła II 24, 60-965 Poznan, Poland; hubert.jopek@put.poznan.pl (H.J.); eli.idczak@gmail.com (E.I.); 2 Institute of Molecular Physics, Polish Academy of Sciences M. Smoluchowskiego 17, 60-179 Poznan, Poland; kww@ifmpan.poznan.pl

**Keywords:** auxetic, negative Poisson’s ratio, honeycomb, re-entrant honeycomb, topology optimization, anomalous properties, computer simulations

## Abstract

This paper presents a finite-element analysis of honeycomb and re-entrant honeycomb structures made of a two-phase composite material which is optimized with respect to selected parameters. It is shown that some distributions of each phase in the composite material result in the counter-intuitive mechanical behaviour of the structures. In particular, negative values of effective Poisson’s ratio, i.e., effective auxeticity, can be obtained for a hexagonal honeycomb, whereas re-entrant geometry can be characterized by positive values. Topology optimization by means of the method of moving asymptotes (MMA) and solid isotropic material with penalization (SIMP) was used to determine the materials’ distributions.

## 1. Introduction

A hexagonal pattern of honeycomb structures has been admired since antiquity. Many centuries ago, Marcus Terentius Varro wrote about the hexagonal shape of bee honeycomb pointing out two possible theories to explain such a structure. One theory held that the hexagons better accommodated the bees’ six feet, while the other, supported by the mathematicians, was that the structure was explained by the isoperimetric property of the hexagonal honeycomb. The honeycomb conjecture states that a regular hexagonal grid or honeycomb is optimal for dividing a surface into equiareal regions with the least total perimeter. The conjecture was proven in 1999 by mathematician Thomas C. Hales [1]. Nowadays, the honeycomb structure is widely used by engineers and scientists in numerous applications including the automotive and aerospace industries etc. A group of scientists from Karlsruhe Institute of Technology in Germany, with the use of laser lithography, manufactured a solid material whose density is lower than the density of water while its tensile strength reaches 280 MPa. The internal structure of this material was based on a honeycomb geometry and several examples of this structure were investigated [2]. Masters and Evans [3] presented a theoretical model for predicting the elastic constants of honeycombs based on the deformation of the honeycomb cells by flexure, stretching and hinging. A few different types of honeycomb system with different mechanical properties have been proposed so far. Grima et al. [4] proposed a new hexagonal honeycomb structure composed of a re-entrant feature (auxetic behaviour) and regular hexagonal honeycombs (conventional behaviour). This new structure has been called a “semi re-entrant honeycomb” and is built of alternate conventional and auxetic layers.

Some first examples of materials and structures characterized by a negative value of the Poisson’s ratio (PR) were presented by Almgren [5] in the case of the two- and three-dimensional structures of hinges, springs and sliding collars. Re-entrant honeycomb structures were discussed by Kolpakov [6]. Gibson and Ashby [7,8] analysed the mechanical properties of two-dimensional cellular materials or honeycombs. Analytical results were compared with experiments. The properties were described in terms of the bending, elastic buckling and plastic collapse of the beams that make up the cell walls. Lakes [9] manufactured auxetic open cell foams. The assemblages of particles linked by springs resulting in a negative Poisson’s ratio was discussed by Evans et al. [10]. Wojciechowski [11] rigorously solved a two-dimensional lattice model of hexagonal molecules which exhibit a negative Poisson’s ratio at high densities. The study confirmed the existence of thermodynamically stable, isotropic, molecular phases of the negative Poisson’s ratio, first observed some years earlier [12] in computer simulations. Using both serial and parallel computer codes Wm. G. Hoover and C. G. Hoover made simulations of auxetic behavior using dynamic analyses of mesoscopic model structures [13]. A comprehensive review of auxetic structures was presented by Lim [14,15]. Auxetic behaviour in two-dimensional ligament structures was studied by Strek et al. [16] for linear elastic and hyperelastic materials. An anlysis of non-linear honeycomb cellular structure was also considered by Cricrì [17]. An analysis of four cylinder-ligament honeycomb structures that form either a hexagonal or re-entrant hexagonal cellular network were described by Alderson et al. [18].

The well-known auxetic structures [19] can be single molecules, crystals, or particular structures of macroscopic or other scale (re-entrant structures, chiral structures, rotating rigid/semi-rigid units, angle-ply laminates, hard molecules, microporous polymers, and liquid crystalline polymer etc.). Re-entrant structures were firstly suggested by Gibson et al. [7]. There are different re-entrant structures summarized and described in the review work of Liu and Hu [19]. Based on their shape, they were named: (a) lozenge grids; (b) sinusoidal ligaments; (c) square grids; (d) double arrowhead; and (e) the structurally hexagonal re-entrant honeycomb.

Strek, Pozniak and co-authors investigated a two-dimensional isotropic material forming a square sample with two sides fixed and the other two remaining under uniform compression load [20,21,22]. It has been shown that negative Poisson’s ratios close to minus one in the corners of the sample behave in a counterintuitive way. The material in those domains moves in the direction opposite to the pressure applied, what can be recognized as a locally negative compliance. The influence of the Poisson’s ratio of a one-side-fixed obstacle on the flow in a two-dimensional channel was studied by a finite-element method (FEM) by Strek et al. [23].

Apart from auxetic structures that form lattices or foams built of just one material, some researchers also investigated more complex structures that are bi- or multi-phase composites. It has been shown by Evans [24] that it is possible to obtain composite material that exhibits a negative Poisson’s ratio if the ratio of the reinforcement to the matrix modulus is sufficiently large. Similar results were presented by Milton [25] who showed that it is possible to create elastically isotropic two- and three-dimensional composites with a Poisson’s ratio approaching −1. Other examples of auxetic composites in which the whole volume is filled with the material were presented by Strek et al. [26,27] and Pozniak [28]. The auxetic composite system built of a conventional honeycomb framework with pores filled with a much softer matrix was investigated by Grima et al. [29]. Analyses of mechanical behaviour of auxetic composites under different conditions e.g., bending, torsion and harmonic loading, were presented by Strek [30,31,32], Jopek [33,34] and Lim [35].

Considering auxetic materials and composites as periodic cellular structures, it is a common approach of researchers to apply homogenization techniques in order to investigate the mechanical properties of materials on the basis of a selected representative unit cell. Kochmann and Venturini [36] analysed the homogenized mechanical properties of two-phased auxetic composite. Gilat et al. used an original homogenization method in order to determine the full tensor of elasticity for the effective continuum [37]. Dirrenberger et al. investigated the behaviour of auxetic structures characterized with anisotropic elastic properties [38,39] and optimized auxetic structures [40] using numerical homogenization. Cellular auxetics with the use of the homogenization technique were also studied by Bacigalupo and Gambarotta [41] and Berinskii [42].

The efficient use of materials is important in many different settings. The optimization of the geometry and topology of the structural layout has a great impact on the performance of structures, and in the last few decades, a great amount of work in this important area of structural optimization has been published [43,44]. These works have mainly been targeted at the material distribution method for generating the optimal topologies of structural elements. Topology optimization has been used by Thomsen to design sandwich-like structures built of one or two materials [45], and Larsen [46] who described a way to design and fabricate compliant micromechanisms and material structures with a negative Poisson’s ratio. Sigmund presented a class of two-phase isotropic composites with extremal bulk modulus [47]. Studying this class of microstructures in their limit of low-volume fractions, the 2D microstructures become rod-like honeycomb structures and the 3D microstructures become closed thin-walled cells. Gibiansky and Sigmund analytically and numerically studied isotropic elastic composites made of three or more isotropic phases [48]. In the 2D case, the optimal microstructures consist of convex disjoint polygonal domains of pure soft or stiff phase connected by laminated plates and transversely isotropic rods made of the two material phases. Schwerdtfeger et al. [49] focused on designing elastic metallic auxetic structures operating through compliant mechanisms with mesoscopic geometric elements in the range of millimetres. Kaminakis and co-authors [50,51] used topology optimization method to design the auxetic microstructures of materials. The methodology of the design was based on a combination of a finite element method and evolutionary-hybrid algorithms. Lukasik [52] presented a new algorithm of the numerical inverse homogenization for the planar isotropic composites with the use of the hexagonal cell of periodicity instead of a rectangular cell.

Czarnecki and co-authors [53,54,55,56] focused on the problem of manufacturability of the minimum compliance designs of the structural elements made of two kinds of inhomogeneous materials: the isotropic and cubic. The material properties have been rationally designed by the isotropic material design (IMD) and the cubic material design (CMD) methods. The authors emphasize that a very important feature of the non-homogeneous solutions, calculated by the IMD method, is the emergence of sub-domains where the Poisson’s ratio is negative and obtainable in the whole range of auxetic isotropic material from −1 to 0 [57]. Andreassen et al. [58] presented a method of designing manufacturable extremal elastic materials with the use of topology optimization. Extremal materials can exhibit interesting properties like, for example, a negative Poisson’s ratio.

Optimized elastic three-dimensional materials with periodic microstructures can be directly manufactured without any need for post-processing by additive manufacturing techniques such as selective laser sintering [59] or laser interference lithography [60].

A tunable material is a material with a variable response to a changing environment or loading that affect this material, e.g., temperature, mechanical forces or displacement, magnetic or/and electric field, light, acoustic wave etc. One can observe a great increase in the interest of researchers in such materials [61].

Tunable mechanical properties of materials composed of fullerene-like spheroids and disordered graphene layers were investigated by Zhao et al. [62]. Lakes, Grima, Ha and others studied the tunable properties of auxetic materials and other smart materials [63,64,65,66]. Li et al. investigated a unit cell with a tunable Poisson’s ratio from positive to negative [67] and a bi-material structure with PR tunable from positive to negative via temperature control [68].

This paper is an extension of the previous works of the present authors. Strek et al. [69] presented numerical results of optimization of a three-layer sandwich two-phase composite. Topology optimization techniques were used for minimization of the effective Poisson’s ratio of the core using a solid isotropic material with the penalization (SIMP) interpolation method of material distribution and the method of moving asymptotes (MMA) as the optimization algorithm. The resultant orthotropic composite structure exhibits a negative Poisson’s ratio, although all its constituents are characterized by positive values of the Poisson’s ratio. Idczak and Strek [70] showed that distribution of reinforcement hard material inside a soft matrix material in the anti-tetra-chiral domain influenced the mechanical properties of the structure. The calculations showed that the resultant structure has a negative Poisson’s ratio even eight times smaller than a homogenous anti-tetra chiral structure made of a common material.

In this paper, the problem of influence of the distribution of two materials on properties of the two-phase hexagonal structure is analyzed. Two hexagonal structures are analyzed: the honeycomb structure and the re-entrant honeycomb structure. The goal of this paper is to show that the distribution of two different materials in two-phase hexagonal structure can *qualitatively* change the mechanical properties of the two-phase hexagonal structure. The distribution of materials in both analyzed structures are obtained with the use of the topology optimization method (using the SIMP and MMA methods) and a finite-element method. It is shown that a honeycomb structure made of two materials can change its Poisson’s ratio from positive to negative. It is also shown that a re-entrant two-phase honeycomb structure can change its Poisson’s ratio from negative to positive. These changes depend on the distribution of both materials and their mechanical properties (Young’s modulus and Poisson’s ratio). The advanced modern structures obtained are characterized by the dependence of their mechanical properties on the mechanical properties of the two building materials. Changing the properties of one material in the two-phase structure one can freely change the Poisson’s ratio of the whole structure.

This unprecedented change in the properties of the honeycomb and re-entrant structures allows for the achievement of new tunable structures.

## 2. Mathematical Formulation of the Problem

### 2.1. Properties of Basic Hexagonal Cellular Structure

Mechanical linear elastic properties (Young’s moduli and Poisson’s ratios) of the doubly-symmetrical two-dimensional hexagonal cellular structure are defined as follows [7]:

(1) $$\begin{array}{cc} E_{H1}= & E_{s}\frac{t^{3}}{l^{3}}\frac{cos\left(\theta \right)}{\left(h/l+\sin\left(\theta \right)\right)sin^{2}\left(\theta \right)}\\ E_{H2}= & E_{s}\frac{t^{3}}{l^{3}}\frac{\left(h/l+\sin\left(\theta \right)\right)}{cos^{3}\left(\theta \right)}\\ \nu_{H12}= & \frac{cos^{2}\left(\theta \right)}{\left(h/l+\sin\left(\theta \right)\right)sin\left(\theta \right)}\\ \nu_{H21}= & \frac{\left(h/l+\sin\left(\theta \right)\right)sin\left(\theta \right)}{cos^{2}\left(\theta \right)} \end{array}$$

where $E_{H1}$, $E_{H2}$,$\;\nu_{H12}$, $\nu_{H21}$ are Young’s moduli and Poisson’s ratios of the hexagonal structure in two orthogonal directions; $E_{s}$ is the wall’s material Young’s modulus; *t* is the thickness of cellular structure walls modelled as a beam; *l* and *h* are the lengths of two joined beams; and θ is angle between two joined beams reduced by 90°. The values of θ and h/l are cell shapes; and t/l is the density of a cell.

Linear elastic properties of regular hexagonal structures (for $\theta =30^{o}$ and h/l=1) simplify as follows [7]:

(2) $$E_{H1}=E_{H2}=\frac{4}{\sqrt{3}}E_{s}\frac{t^{3}}{l^{3}}\;\;\mathrm{and}\;\nu_{H12}=\nu_{H21}=1$$

These properties confirm that a regular hexagonal structure is isotropic, and a doubly-symmetrical two-dimensional hexagonal cellular structure is orthotropic.

### 2.2. Topology Optimization of Two-Phase Structures

Topology optimization (TO) is a mathematical method that optimizes material or materials’ layout within a defined design domain, for a given set of loads and constraints [43,44]. One can describe the performance of the system using a mathematical formula with given set of parameters (goal function). Optimization of the system can be attained by maximizing or minimizing the goal function. In general, the design can attain any shape within the design space, instead of dealing with predefined configurations from shape optimization [71].

The classic formulation uses a finite-element method to evaluate the design performance. The design is optimized using either gradient-based mathematical programming techniques (e.g., the optimality criteria algorithm, the method of moving asymptotes) or gradient-free algorithms (e.g., genetic algorithms, a differential evolution algorithm).

Optimization in mechanical design is a process of finding such shapes of constructions or material distribution that allow the system to fulfil certain earlier assumed criteria. Practically, it is not efficient or even not necessary to search for the global optimum. Given that, many sub-optimal solutions fulfil the assumed criteria and it is possible to obtain different solutions depending on the optimization method used. Hence, the optimization problem is determined not only by the goal function and criteria but also by the method applied. One of the most popular methods used in optimization of materials distribution problems is a SIMP method. An analysis of effective properties of the two-phase material is based on methods of evaluation of generalized properties such as Young’s modulus, Poisson’s ratio and density. These generalized properties are expressed by means of the SIMP scheme. The main part of this scheme is based on the interpolation function that represents various generalized physical quantities as a function of a continuous variable *r*. The parameters in the SIMP method fulfil the equations:

(3) $$E\left(r\right)=E_{1}+\left(E_{2}-E_{1}\right)\cdot r^{p}$$

where *r = r*(*x*) is the control variable and 0 < *r* < 1; *p* is the penalization parameter; and *E*_1_ and *E*_2_ are Young’s moduli for the first and the second material respectively (*E*_1_ ≤ *E*_2_).

In all cases considered, both materials differ with each other only by the values of Young’s modulus. The interpolating function (3) is used to describe the distribution of materials with different properties inside the analysed domain.

The most common definition of Poisson’s ratio is based on the assumption of small deformation and it is computed as a negative ratio of the average transverse to longitudinal strains:

(4) $$\nu_{eff}=-\frac{\bar{\varepsilon_{t}}}{\bar{\varepsilon_{l}}}$$

where: $\bar{\varepsilon_{t}}$ is the average strain in the transverse direction; and $\bar{\varepsilon_{l}}$ is the average strain in the longitudinal direction. For a given anisotropic material, the value of the Poisson’s ratio is a function of the selected direction in which the loading is applied [39]. In this case, the direction is specified: the force is applied along the *y*-axis, so the average transverse strain is defined by the equation:

(5) $$\bar{\varepsilon_{t}}=\frac{\int_{G_{1}}u_{1}dG}{L_{x}\cdot \int_{G_{1}}dG}$$

where: *G*_1_ is the boundary parallel (*x* = *L_x_*) to the boundary with applied prescribed displacement; and *u*_1_ is displacement in the *x*-axis. The average longitudinal strain is defined by the equation:

(6) $$\bar{\varepsilon_{l}}=\frac{\int_{G_{2}}u_{2}dG}{L_{y}\cdot \int_{G_{2}}dG}$$

where: *G*_2_ is the boundary parallel (*y* = *L_y_*), where a load is applied; and *u_2_* is displacement in the *y*-axis. Because of the use of the SIMP scheme, the effective Poisson’s ratio must be dependent on the control variable *r*. In this way, the equation receives objective functions for the optimization problem of the two-phase chiral shape:

(7) $$\nu_{eff}\left(r\right)=-\frac{\bar{\varepsilon_{t}\left(r\right)}}{\bar{\varepsilon_{l}\left(r\right)}}$$

The control variable function of the SIMP scheme is constrained by two conditions: pointwise inequality (8) and integral inequality (9), which are given by formulae:

(8) 0≤r(x)≤1 for x∈S

(9) $$0\leq \int_{S}r\left(x\right)dS\leq S\cdot A_{f}$$

where *x* is defined coordinate; and *A_f_*—is a fraction of the second material in the domain *S*. The process of optimization is defined in the following order: first FEM—discretization, the redefinition of function minimization with applied constraints as a standard finite-dimensional non-linear programmable problem. Then the value of the control variable is evaluated in every mesh node as:

(10) $$r\left(x\right)=\overset{N}{\underset{i=1}{\sum }}r_{i}\cdot \phi_{i}\left(x\right)$$

where: *φ_i_*(*x*) are shape functions and *N* is the number of an element’s node.

After discretization, the pointwise inequality condition is expressed as follows:

(11) $$0\leq r_{i}\leq 1\;for\;x=1,\ldots ,M$$

where *M* is the number of all nodes of all mesh elements in which the values of the control variable are calculated.

### 2.3. Numerical Model

The unit cell of a honeycomb structure is typically characterized by positive values of the Poisson’s ratio if the cell is made of a homogeneous isotropic material. On the contrary, the re-entrant unit cell is known as a typical auxetic shape; i.e., its Poisson’s ratio of such structure is negative if it is made of homogeneous isotropic material. However, it is possible to build these structures using a composite material which is neither homogeneous nor isotropic. In such a case, effective properties of the resultant structure may significantly differ from basic ones.

The isotropic materials’ parameters of composite constitutes used in the simulation are:

- for soft material (matrix material): Young’s modulus $E_{1}$ = {$10^{8}$, $10^{9}$} Pa, and the Poisson’s ratio is $\nu_{1}=0.33$;
- for hard material (reinforcement material): Young’s modulus $E_{2}=10^{11}$ Pa and the Poisson’s ratio is $\nu_{2}=0.33$.

So, two cases are considered: the ratio of the Young’s moduli of materials, $RE=E_{2}/E_{1}$, equals $10^{2}$ or $10^{3}$.

**Figure 1:**
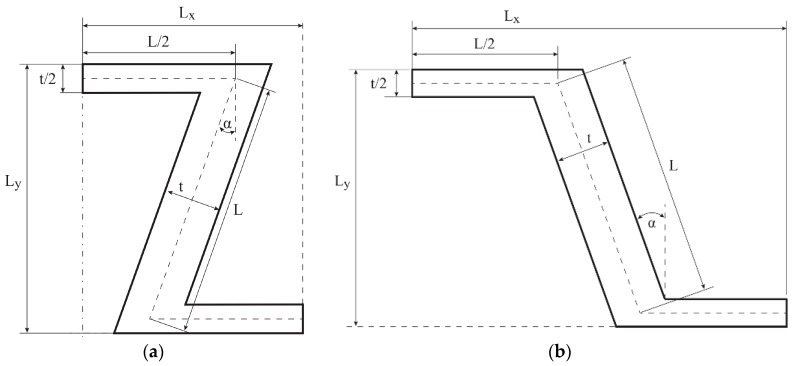

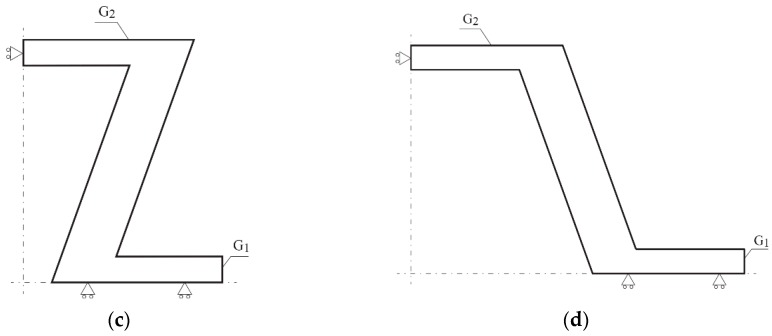
The geometry of the quarter of: (**a**) re-entrant honeycomb; (**b**) honeycomb; boundary conditions for: (**c**) re-entrant honeycomb; (**d**) honeycomb geometries.

In all cases, the structure is compressed. Boundary $G_{2}$ is moved down by 0.01 m (see Figure 1c,d).

Also, two values of the parameter *t* are considered, which means that the thickness of the ribs equals either 0.2 or 0.28. In all cases, the value of the rib length *L* was constant and resulted from the length of the side of a regular hexagon inscribed in the circle of radius equalling 1. On the basis of the length *L* and the angle *α* = 15°, the dimensions of the cell *L_x_* and *L_y_* are calculated. All these values are collected in Table 1.

All numerical calculations were performed by means of FEM software (Comsol Multiphysics, Stockholm, Sweden). The finite-element mesh consisted of over 29,000 triangle elements for the thickness of ribs equal to 0.20 and over 40,000 for thickness equal to 0.28 (maximum mesh element size was 0.005). Optimization control variable shape functions were defined as bilinear Lagrange polynomials, while quadratic Lagrange polynomials were used as shape functions for displacement field components in-plane stress Navier’s equation. The value of the penalization parameter of the SIMP model is *p* = 3.

FEM together with the method of moving asymptotes (MMA) was used to find the optimal distribution of two positive materials in the composite. The MMA algorithm [72,73] is based on the idea of using a shifted Lagrangian function to make a convex approximation and then solves a sequence of convex approximations. The MMA algorithm is used to solve the optimization problem, which allows determination of the distribution of two materials in the considered domain. One can obtain this by using the MMA to minimize the objective function defined as the effective Poisson’s ratio of the structure with some proper constraints.

## 3. Results

In this section, we present the results of the topology optimization used in order to distribute constituents in the composite materials so that the effective Poisson’s ratios of considered geometries are opposite to those obtained in the case of homogeneous isotropic material. So, in the case of a honeycomb structure, the goal of the optimization process was to find such distribution for which the effective Poisson’s ratio is minimal (negative if attainable); whereas in the case of re-entrant geometry, the optimization process was used in order to determine such distribution of composite materials that results in the maximum value of the effective Poisson’s ratio (positive if attainable).

The plots of topological optimization results are compiled in tables in order to simplify the comparison. Composite structures obtained for honeycomb geometry are presented in Table 2, and structures obtained for re-entrant geometries are presented in Table 3. Due to small values of deformation obtained in some cases, all deformations presented in the figures below are magnified by factor 5. In the case of honeycomb geometries, negative values of the effective Poisson’s ratio are obtained by such material distribution in which the hard material creates trusses nearby the joints of ribs, and these trusses are joined in such a way that, overall, the hard material in fact creates a re-entrant pattern inside a convex hexagon sample cell. Hence, the honeycomb structure acts like an auxetic one, and so its effective PR (Poisson’s ratio) value is negative.

The *A_f_* parameter that describes the share of hard material in the sample cell volume *ceteris paribus* does not change significantly the way in which both materials are distributed in the considered domain. However, in the case of a greater amount of the hard material, the latter reinforces mainly the trusses in the joints. A greater share of hard material in most cases enhances the effect of optimization; i.e., lower negative values of *ν_eff_* in the case of honeycomb geometry and higher positive values of the *ν_eff_* in the case of re-entrant geometry. For example, geometries presented in Table 2 at row 1, column A (1A) and row 3, column A (3A) are very much alike; all parameters are the same apart from *A_f_* and the value of *ν_eff_* = −0.78 in the case of *A_f_* = 20% and *ν_eff_* = −1.41 in the case of *A_f_* = 40%.

Re-entrant honeycomb composite structures are optimized in order to maximize the value of their effective Poisson’s ratio, possibly to obtain positive values.

The hard material is distributed in a re-entrant honeycomb composite so that it forms a shape similar to a convex polygon. The polygon can be created within the re-entrant honeycomb structure if the amount of hard material is sufficient and the geometrical parameters *t* and angle *α* are such that a convex polygon can be inscribed into the shape of the cell. Such distribution occurs in all cases in which *t* = 0.28, but it is not possible in the case of *t* = 0.2. In general, the material distribution is such that the structure that is formed by the hard phase allows for the deformation mechanism similar to that in the typical honeycomb.

When *t* = 0.2, the stiffer material tends to concentrate in the corners of the cell in which it creates truss-like structures connected with each other, with long “beams” that run along the side walls of the cell. These truss-like structures reinforce the corners, preventing the cell from collapsing, which is typical behaviour for the re-entrant cell. The beams that occur in the side walls of the cell are directed outwards and so they expand the cell while compressed even though the geometry of the whole cell is re-entrant. Additionally, the truss-like structures and beams are connected with each other almost pointwise, like joints, which enables rotations and bending of the cell’s sides.

The parameter that strongly influences the deformation behaviour of the cell is the ratio of Young’s moduli of both phases. The value of the effective Poisson’s ratio $\nu_{eff}$ changes from 1.74 in the case of *RE* = 10^2^ to 12.97 for *RE* = 10^3^, *A_f_* = 0.2 in both cases. Similar change in $\nu_{eff}$ occurs for a structure in which the share of stiffer material is 40% (*A_f_* = 0.4).

In the case of the re-entrant honeycomb cell with the thicker walls, *t* = 0.28, the distribution of both materials is different than that obtained for *t* = 0.2. The stiffer material does not occur in corners at all, but partly reinforces the top and bottom edges of the re-entrant hexagonal cell and creates the beams that run along the walls so the structure takes the form of a convex polygon. The “beams” are also connected pointwise, which allows for rotation about corners, and the deformation mechanism is similar to that of a typical honeycomb. The increase of the stiffer material phase does not change the form of the structure, only the “beams” become thicker. Moreover, the value of the effective Poisson’s ratios are also close to each other in both analysed cases of *A_f_*. which implies that the crucial parameter is *RE*. The increase of the ratio of Young’s moduli naturally enhances the stiffness of “beams”, and so the behaviour of the whole composite is determined mostly by the structure of reinforcement. If the value of *RE* is close to 1, the behaviour of the composite is dependent on the geometrical shape of the whole composite.

All the structures presented in Table 2 and Table 3 show the structures that, under the conditions of adjustable Young’s modulus of the second material or both materials, become tunable structures. In both types of structure, we observe *counter-intuitive* values of Poisson’s ratios.

The classic homogenous honeycomb structure is characterized by a positive Poisson’s ratio. By contrast, the proposed novel two-phase honeycomb composite structures can lead to auxetic behaviour.

On the other hand, an example of the well-known auxetic structure is a homogenous re-entrant structure characterized by a negative Poisson’s ratio. In the way presented in this paper, the novel two-phase re-entrant composite structure can generate a positive Poisson’s ratio.

For further consideration, two exemplary composite structures were selected for analysis. The first is based on the construction of a classic honeycomb geometry and the second on re-entrant geometry. In both the tunable, two-phase composite structures (honeycomb and re-entrant), the second material occupies 20% of the total structure and the thickness of ribs (walls) is *t* = 0.2. The distributions of materials for both the tunable composites are presented in Table 2 (2A) for the honeycomb composite, and in Table 3 (2A) for the re-entrant composite.

The value of the Young’s modulus of the second material can change from 10^8^ to 10^11^ Pa, which results in values of the ratio of Young’s moduli RE that vary in the range from 1 to 10^3^. Both the structures are built from homogeneous ribs for *RE* = 1 ($E_{1}=E_{2}=10^{8}$) and non-homogeneous for greater values of the ratio RE parameter ($E_{1}<E_{2}$). All two-phase composite structures considered are anisotropic.

In the case of the two-phase honeycomb composite structure, the effective Poisson’s ratio changes from +2.3 for $E_{2}=10^{8}$ to −7.2 for $E_{2}=10^{11}$. In the second case, of the two-phase re-entrant composite structure, the effective Poisson’s ratio changes from −4.1 for $E_{2}=10^{8}$ to +13.6 for $E_{2}=10^{11}$.

**Figure 2:**
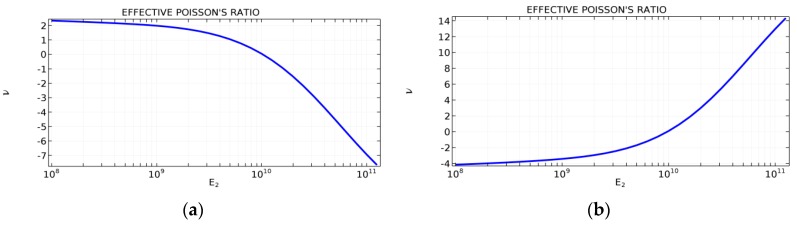
Values of the effective Poisson’s ratio (PR) for different values of $E_{2}$ of tunable two-phase (**a**) honeycomb; and (**b**) re-entrant honeycomb composite structures.

The sign of effective Poisson’s ratio changes for $E_{2}$ equal to about $10^{10}$ ($\mathrm{RE}≅10^{2}$) (see Figure 2) for both composite structures. In the case of honeycomb structures, the sign changes to minus, while in the case of re-entrant structures, the sign changes to plus one.

## 4. Conclusions

The study presented in this paper shows that by embedding reinforcements of a hard material in the soft material of well-known (auxetic or non-auxetic) structure one can *qualitatively* change its elastic properties. In this paper, this change was proven by examples of samples in which hard material fills in 20% or 40% of the structure domain. These examples showed that the well-known honeycomb structure, typically characterized by a positive Poisson’s ratio, can change its properties and behave like a structure with a negative Poisson’s ratio. For analogous reasons, re-entrant honeycomb structures, typically with negative PR, may “lose” their auxeticity and transform themselves into structures exhibiting positive PR. In both cases presented in this paper, the transformed structures presented are anisotropic.

## Figures and Tables

**Table 1:** The values of geometrical characteristics, number of finite elements, and number optimization variables of considered geometries.

-	Geometry 1	Geometry 2	Geometry 3	Geometry 4
Parameter	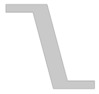	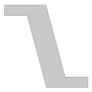	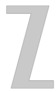	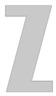
Thickness of ribs *t* (m)	0.20	0.28	0.20	0.28
*L_x_* (m)	1.1248	1.1248	0.60721	0.60721
*L_y_* (m)	1.0659	1.1059	1.0659	1.1059
Area (m^3^)	0.2866	0.40124	0.2866	0.40124
Number of mesh elements	29,014	40,766	29,420	40,974
Number of optimization variables	14,902	20,786	15,106	20,890

**Table 2:** Results of honeycomb topology optimization. Parameters of each case, the optimized distribution of constituents, and the deformed shape of a vertically compressed shape.

-	-	A	B
No.	Parameters	The optimized distribution of constituents (hard material—blue, soft material—green)	The deformed shape and the displacement field (colour map represents the values of total displacements (m)).
1	*RE* = 10^2^ *A_f_* = 0.2 *t* = 0.2 *ν_eff_* = −0.78	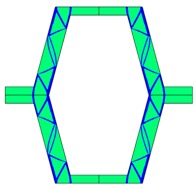	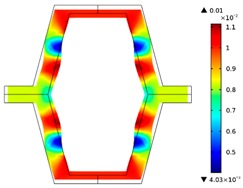
2	*RE* = 10^3^ *A_f_* = 0.2 *t* = 0.2 *ν_eff_* = −6.91	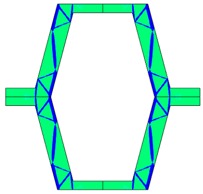	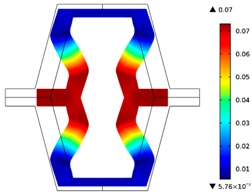
3	*RE* = 10^2^ *A_f_* = 0.4 *t* = 0.2 *ν_eff_* = −1.41	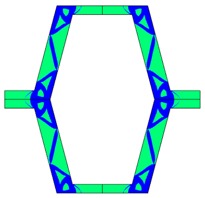	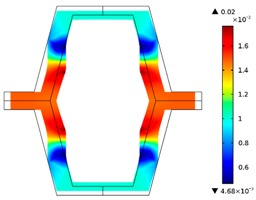
4	*RE* = 10^3^ *A_f_* = 0.4 *t* = 0.2 *ν_eff_* = −7.95	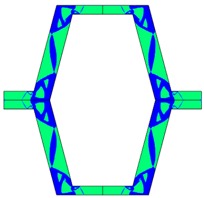	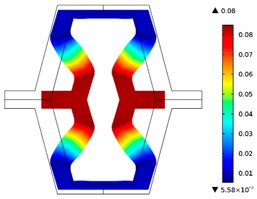
5	*RE* = 10^2^ *A_f_* = 0.2 *t* = 0.28 *ν_eff_* = −1.89	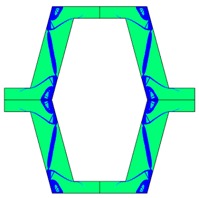	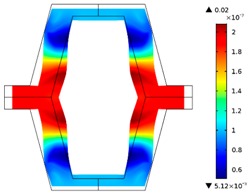
6	*RE* = 10^3^ *A_f_* = 0.2 *t* = 0.28 *ν_eff_* = −10.33	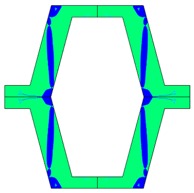	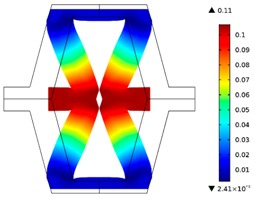
7	*RE* = 10^2^ *A_f_* = 0.4 *t* = 0.28 *ν_eff_* = −2.39	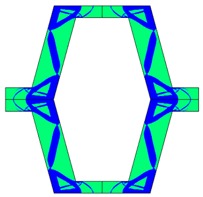	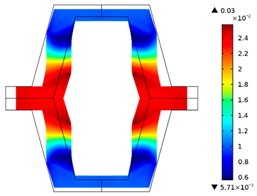
8	*RE* = 10^3^ *A_f_* = 0.4 *t* = 0.28 *ν_eff_* = −9.17	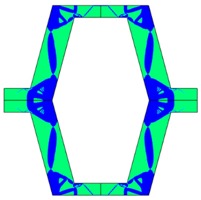	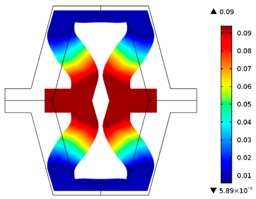

**Table 3:** Results of re-entrant honeycomb topology optimization. Parameters of each case, the optimized distribution of constituents, and the deformed shape of a vertically compressed shape.

-	-	A	B
No.	Parameters	The optimized distribution of constituents (hard material—blue, soft material—green)	The deformed shape and the displacement field (colour map represents the values of total displacements (m)).
1	*RE* = 10^2^ *A_f_* = 0.2 *t* = 0.2 *ν_eff_* = 1.74	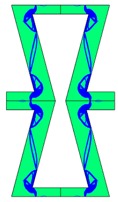	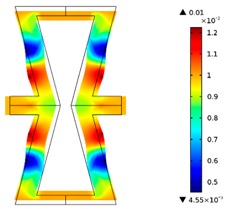
2	*RE* = 10^3^ *A_f_* = 0.2 *t* = 0.2 *ν_eff_* = 12.97	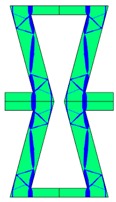	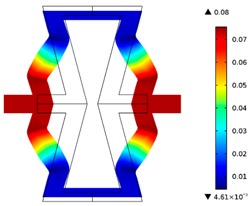
3	*RE* = 10^2^ *A_f_* = 0.4 *t* = 0.2 *ν_eff_* = 2.95	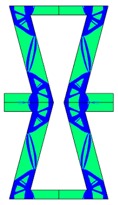	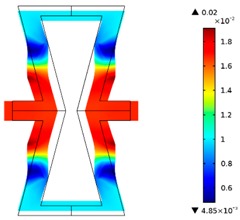
4	*RE* = 10^3^ *A_f_* = 0.4 *t* = 0.2 *ν_eff_* = 16.11	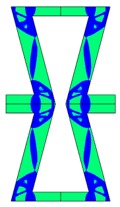	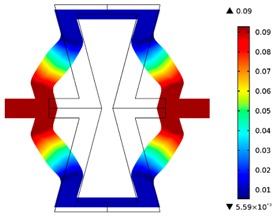
5	*RE* = 10^2^ *A_f_* = 0.2 *t* = 0.28 *ν_eff_* = 8.42	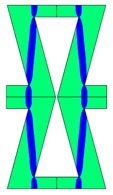	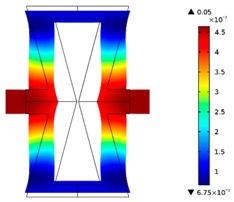
6	*RE* = 10^3^ *A_f_* = 0.2 *t* = 0.28 *ν_eff_* = 27.84	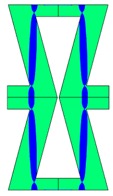	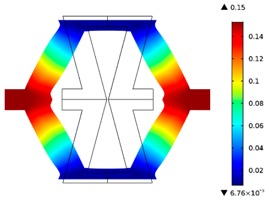
7	*RE* = 10^2^ *A_f_* = 0.4 *t* = 0.28 *ν_eff_* = 9.67	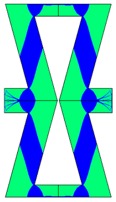	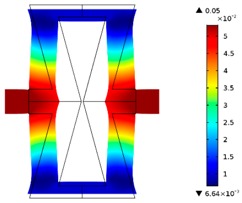
8	*RE* = 10^3^ *A_f_* = 0.4 *t* = 0.28 *ν_eff_* = 29.7